# STUDENTS CONNECTING THE DOTS BETWEEN POLICY THROUGH SERVICE, RESEARCH, AND TEACHING

**DOI:** 10.1093/geroni/igae098.1394

**Published:** 2024-12-31

**Authors:** Shannon Jarrott

**Affiliations:** The Ohio State University, Columbus, Ohio, United States

## Abstract

Students pursuing social sciences degrees because of a desire to improve individual, family, and community circumstances should learn how policy impacts their efforts. I offer examples of teaching, research, and service that orient students to gerontological policy at the organizational, state, and national levels. Examples illustrate fortitude of students, older adults, and organizations in the face of emergent need and opportunity. At the organizational level, students engaged in service at an intergenerational day program for children and older adults developed a satellite food pantry in response to intergenerational need for healthy food access. At the state level, the Ohio the Scholars in Aging program convenes annual cohorts of undergraduate and graduate students from across the state in study of policy making via a partnership between the Ohio Association for Gerontology Education (OAGE) and the Ohio Department of Aging. At the federal level, research students facilitated older adults’ access to the federally funded Senior Farmers’ Market Nutrition Program during COVID. Students’ goals to support the well being of older adults and their families and communities are facilitated through engaged learning, service, and research that connects theory, skills, evidence, and policy. Students may learn about policies that are responsive to emergent need and can harness resources across agencies. They may also identify policies that isolate organizations and prevent collaboration, even when addressing similar goals for different populations. Awareness of the different shapes policies take in their objectives, implementation, and impact enhances students’ ability to navigate and utilize policy to positive effect.

